# Turbo Decoder Design based on an LUT-Normalized Log-MAP Algorithm

**DOI:** 10.3390/e21080814

**Published:** 2019-08-20

**Authors:** Jun Li, Xiumin Wang, Jinlong He, Chen Su, Liang Shan

**Affiliations:** 1 Binjiang College, Nanjing University of Information Science & Technology, Wuxi 214105, China; 2 College of Information Engineering, China Jiliang University, Hangzhou 310018, China

**Keywords:** turbo decoder, normalized-Log-MAP algorithm, normalization functional unit, LTE-advanced, cyclone IV

## Abstract

Turbo codes have been widely used in wireless communication systems due to their good error correction performance. Under time division long term evolution (TD-LTE) of the 3rd generation partnership project (3GPP) wireless communication standard, a Log maximum a posteriori (Log-MAP) decoding algorithm with high complexity is usually approximated as a lookup-table Log-MAP (LUT-Log-MAP) algorithm and Max-Log-MAP algorithm, but these two algorithms have high complexity and high bit error rate, respectively. In this paper, we propose a normalized Log-MAP (Nor-Log-MAP) decoding algorithm in which the function max* is approximated by using a fixed normalized factor multiplied by the max function. Combining a Nor-Log-MAP algorithm with a LUT-Log-MAP algorithm creates a new kind of LUT-Nor-Log-MAP algorithm. Compared with the LUT-Log-MAP algorithm, the decoding performance of the LUT-Nor-Log-MAP algorithm is close to that of the LUT-Log-MAP algorithm. Based on the decoding method of the Nor-Log-MAP algorithm, we also put forward a normalization functional unit (NFU) for a soft-input soft-output (SISO) decoder computing unit. The simulation results show that the LUT-Nor-Log-MAP algorithm can save about 2.1% of logic resources compared with the LUT-Log-MAP algorithm. Compared with the Max-Log-MAP algorithm, the LUT-Nor-Log-MAP algorithm shows a gain of 0.25~0.5 dB in decoding performance. Using the Cyclone IV platform, the designed Turbo decoder can achieve a throughput of 36 Mbit/s under a maximum clock frequency of 44 MHz.

## 1. Introduction

Turbo codes were proposed in 1993, and have been widely used in 3G and 4G wireless communication systems due to their good error correction [1]. Yang et al. proposed new, partially information-coupled (PIC) turbo codes in 2018 [2], which can improve decoding performance without changing the decoder. The Log-MAP algorithm was often used in decoding turbo codes. In order to reduce logic resource consumption with the Log-MAP algorithm, Martina et al. proposed a turbo decoder with low complexity based on an approximated Log-MAP algorithm in 2014 [3]. Compared with the Log-MAP algorithm, the approximate Log-MAP algorithm can save nearly 30% of logic resources by linear approximation of the function max*. In 2015, Ivanov et al. summarized the common approximation methods of the max* function [4,5]. In 2018, Liu et al. proposed an improved turbo decoder that approximates the function max* with a Taylor series [6]. The idea of normalized approximation mainly comes from the low-density parity check (LDPC) decoder based on the normalized min-sum (NMS) algorithm, as proposed by Wu et al. in 2010 [7], and we use this method to approximate the function max*. In 2011, Sun et al. proposed a dynamic interleaving structure based on a quadratic permutation polynomial (QPP) interleaver that performs real-time interleaving address output and reduces storage unit consumption [8,9]. Sun et al. also proposed a flexible functional unit (FFU) for a soft-input soft-output (SISO) decoder, while a turbo decoder achieves a throughput of 1280 Mbit/s with a clock frequency of 400 MHz under 3GPP-LTE and LTE-advanced standards [9,10]. Rohit et al. put forward an idea for a hybrid algorithm in 2013, and its decoding performance is better than a Log-MAP algorithm after combining a LUT-Log-MAP algorithm and Linear-Log-MAP algorithm in every state metric calculation [11], although this implementation results in a large consumption of logic resources. In 2017, Zhen et al. proposed a calculation method of the function max* that can be applied for reverse calculation in engineering implementation [12].

In this paper, we propose a Nor-Log-MAP algorithm that uses a fixed normalized factor to approximate the function max*. According to the idea of a hybrid algorithm [11], we also propose a LUT-Nor-Log-MAP algorithm that uses different algorithms in different metric calculation states so as to reduce the number of lookup tables and consumption of logical resources. LUT-Nor-Log-MAP is a new kind of algorithm that combines Nor-Log-MAP with LUT-Log-MAP. The LUT-Log-MAP algorithm is used to calculate forward-state and backward-state metrics, and the Nor-Log-MAP algorithm is used to compute external information and posteriori information. Based on the LUT-Nor-Log-MAP algorithm, we incorporated an FFU and a normalization functional unit (NFU) into the design of the SISO unit.

The rest of this paper is organized as follows. Section 2 reviews turbo decoding principles and the Log-MAP algorithm. Section 3 presents the Nor-Log-MAP algorithm. Section 4 introduces the LUT-Nor-Log-MAP algorithm and the structure of the SISO unit. Section 5 analyzes the proposed algorithm. Section 6 presents the synthesis results and comparisons. Section 7 concludes the paper.

## 2. Turbo Decoding Principles and Log-MAP Algorithm

### 2.1. Turbo Decoding Principles

**Figure 1:**
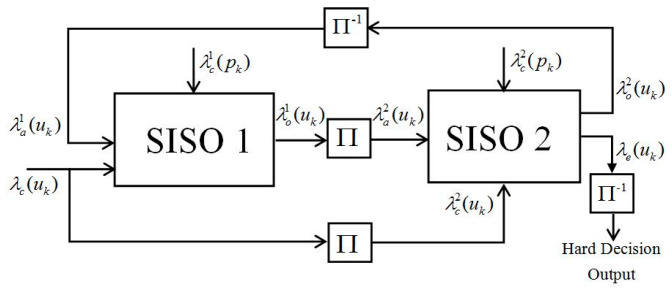
Traditional turbo decoding procedure using a SISO decoder.

The decoding process of turbo codes involves the exchange of soft information between two SISO decoders [10]. In turbo encoding and decoding processes, we use the same interleaver, where de-interleaving is the inverse process of interleaving. The SISO decoder consists of three input ports, which are system information $\lambda_{c}\left(u_{k}\right)$, parity information $\lambda_{c}\left(p_{k}\right)$, and a priori information $\lambda_{a}\left(u_{k}\right)$ which is computed by another SISO decoder. Two output ports of the SISO decoder generate external information $\lambda_{e}\left(u_{k}\right)$ and posteriori information $\lambda_{o}\left(u_{k}\right)$. The different superscripts represent information corresponding to different SISO decoders, and subscript *k* denotes *k*-th bit information of the current variable. Π and $\Pi^{-1}$ denote interleaving and de-interleaving, respectively. When the iteration stopping criterion is satisfied, decoding results can be obtained from $\lambda_{e}\left(u_{k}\right)$ after the de-interleaved and hard-decision operations [11,13]. The turbo decoding procedure is shown in Figure 1.

### 2.2. Log-MAP Algorithm

A priori information is computed by Equation (1), where the initial value of priori information is set to zero for the first iteration [8].

(1) $$\lambda_{a}\left(u_{k}\right)=\log\frac{P\left(u_{k}=1\right)}{P\left(u_{k}=0\right)}$$

In SISO, branch metric $\gamma_{k}$ and extrinsic branch metric $\gamma_{k}^{e}$ are computed by Equations (2) and (3), where *n* is the number of bits at the output of the component convolutional encoder of the turbo code, and *i* denotes the *i*-th bit of codeword [8].

(2) $$\gamma_{k}=u_{k}\cdot (\lambda_{c}(u_{k})+\lambda_{a}(u_{k}))+\sum_{i=1}^{n}p_{k}^{\left(i\right)}\cdot \lambda_{c}(p_{k}^{\left(i\right)})$$

(3) $$\gamma_{k}^{e}=\sum_{i=1}^{n}p_{k}^{\left(i\right)}\cdot \lambda_{c}(p_{k}^{\left(i\right)})$$

Then, we use Equations (4) and (5) to compute the forward and backward metrics recursively, $s_{k}$ and $s_{k-1}$ being the two states on opposite sides of the branch metric.

(4) $$\alpha_{k}(s_{k})={\underset{s_{k-1}}{\max}}^{*}\{\alpha_{k-1}(s_{k-1})+\gamma_{k}(s_{k-1},s_{k})\}$$

(5) $$\beta_{k}(s_{k})=\underset{s_{k+1}}{\max^{*}}\{\beta_{k+1}(s_{k+1})+\gamma_{k}(s_{k},s_{k+1})\}$$

Finally, external information and posteriori information are computed by Equations (6) and (7), and generated posteriori information is taken as the prior information for another SISO decoder in the next iteration.

(6) $$\lambda_{e}(u_{k})=\overset{}{\underset{u_{k}=1}{\max^{*}}}\left\{\alpha_{k-1}(s_{k-1})+\gamma_{k}^{}(s_{k-1},s_{k})+\beta_{k}(s_{k})\right\}\;-\overset{}{\underset{u_{k}=0}{\max^{*}}}\left\{\alpha_{k-1}(s_{k-1})+\gamma_{k}^{}(s_{k-1},s_{k})+\beta_{k}(s_{k})\right\}$$

(7) $$\lambda_{o}(u_{k})=\lambda_{e}(u_{k})-\lambda_{a}(u_{k})-\lambda_{c}(u_{k})$$

The function max* in Equations (4)–(6) is defined as

(8) $$\max^{*}(a,b)=\max(a,b)+\log(1+e^{-|a-b|})$$

The variable *Lach* is defined as Equation (9).

(9) $$Lach=\lambda_{a}(u_{k})+\lambda_{c}(u_{k})$$

## 3. The Normalized Log-MAP Algorithm

The function $\log(1+e^{-\left|a-b\right|})$ is a nonlinear calculation in Equation (8) that will consume lots of logic resources in decoder design, so we need to simplify the function $\log(1+e^{-\left|a-b\right|})$ in max*. In [4,5], two commonly used algorithms for approximating the nonlinear function $\log(1+e^{-\left|a-b\right|})$ are as follows:

(a) The $\max^{*}$ approximation method of the Max-Log-MAP algorithm is shown in Equation (10).

(10) $$\log(1+e^{-|a-b|})\approx 0$$

(b) The max* approximation method of the LUT-Log-MAP algorithm is shown in Table 1 [9].

The Max-Log-MAP algorithm consumes fewer logic resources, but has a poor decoding performance. The LUT-Log-MAP algorithm has a better decoding performance, but consumes a large amount of logic resources. The LUT-Log-MAP algorithm reduces the complexity of the Log-MAP algorithm in hardware implementation with only 0.1 dB decoding performance loss, so it can be said that the performance of the LUT-Log-MAP algorithm is better. However, the LUT-Log-MAP algorithm involves a large number of lookup table operations in hardware implementation, as well as having a large consumption of logical resources. So finding a max* approximation method becomes the key point in obtaining considerable decoding performance and reducing logic resource consumption.

**Figure 2:**
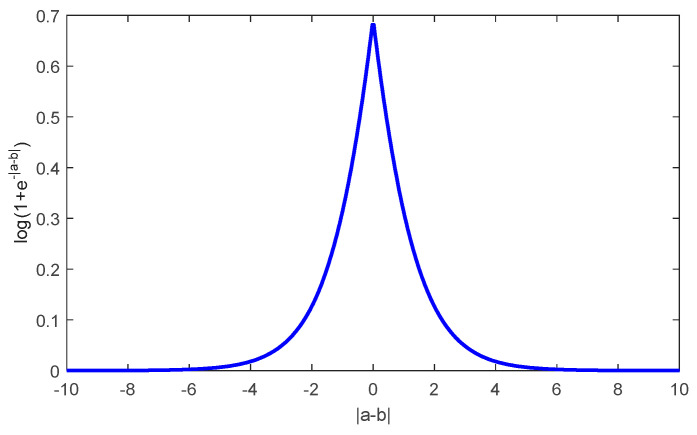
The curve of function $\log(1+e^{-\left|a-b\right|})$.

It can be seen from Figure 2 that function $\log(1+e^{-\left|a-b\right|})$ has a small range of $0<\log(1+e^{-\left|a-b\right|})<0.7$ with arbitrary real numbers a and b. Therefore, we try to establish Equation (11) by finding a fixed normalization factor η (η>1).

(11) $$\max(a,b)+\log(1+e^{-|a-b|})\approx \eta \cdot \max(a,b)$$

From the approximation method of the LUT-Log-MAP algorithm [6], when |a−b|>2, the value of function $\log(1+e^{-\left|a-b\right|})$ approaches zero and becomes the Max-Log-MAP algorithm [7]. A lookup table is used to facilitate the algorithm in hardware implementation. Combining this idea, the proposed Nor-Log-MAP algorithm will determine the value of η under condition |a−b|≤2, and the value of η in |a−b|>2 will be equal to 1. Then, the optimal range |a−b| can be verified under this fixed η. The Nor-Log-MAP algorithm based on the above concept is shown in Equation (12).

(12) $$\max^{*}(a,b)\approx \{\begin{array}{c} \eta \cdot \max(a,b)\\ \max(a,b) \end{array}\begin{array}{c} ,if|a-b|<\mathrm{range}\\ ,\mathrm{otherwise} \end{array}$$

Turbo codes with a 1/2 code rate and 192 code length are used in a MATLAB simulation. As the final result needs to support hardware implementation, all the variables’ values in the algorithm will go through Q2 quantization (Q total bits with 2 fractional bits) [8]. The Q2 quantization involves a decimal number with two fractional bits going through binary conversion, followed by the removal of the fractional bits and converting into a decimal number. Therefore, in the process of simulation, |a−b|≤2 will be |a−b|≤8 by Q2 quantization. We can achieve the lookup table method based on Q2 quantization, as shown in Table 2 [8], and this method is also used in normalized quantization.

**Figure 3:**
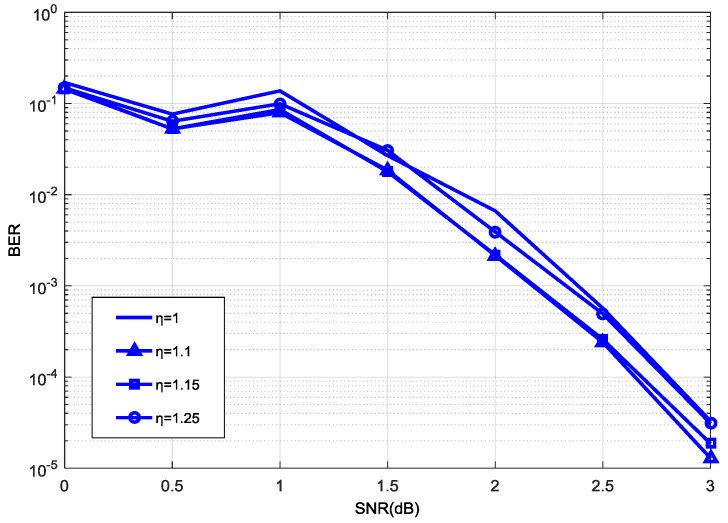
Decoding performance with different values of η (1~1.25).

**Figure 4:**
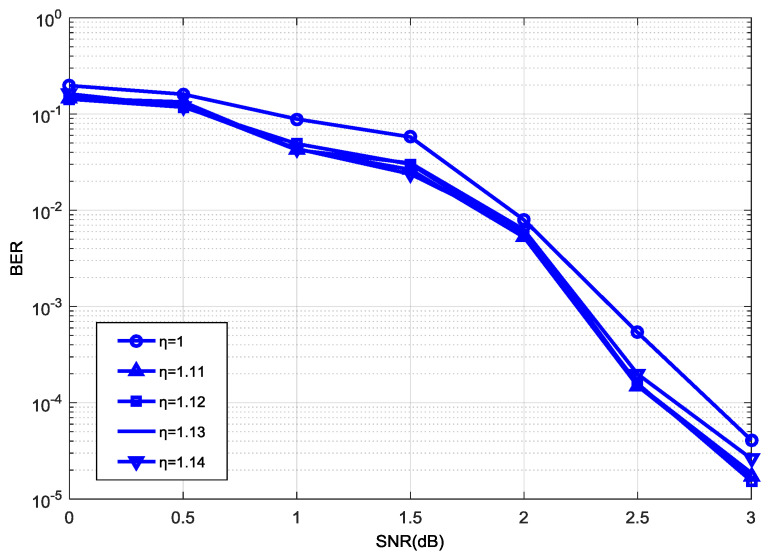
Decoding performance with different values of η (1.11~1.14).

**Figure 5:**
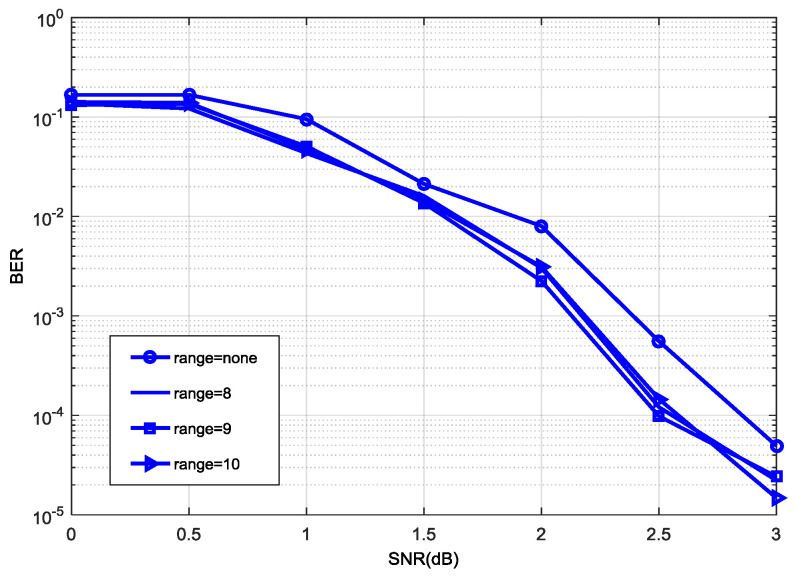
Decoding performance with same η and different ranges.

In order to make Figure 3, Figure 4 and Figure 5 clearer, we omit other poor performance curves. Figure 3 shows the effect of decoding performance with different normalization factors η(1≤η≤1.25) under the range of |a−b|≤8 with a step size of η = 0.05. Figure 3 shows that we can achieve the best decoding performance when η = 1.1, and the decoding performance was low when η was between 1 and 1.25. We set η = 1.1 as the center to find a more accurate η. In Figure 4, we achieved the best decoding performance when η was between 1.11 and 1.14. Considering that addition will reduce more logic resource consumption than multiplication in a hardware simulation, we can change the multiplication of η and the function max* into an addition of the max and its shift value. But addition can only be performed when the fractional part of the normalization factor *η* is $2^{-n}$ ($n\in N^{*}$). Since the fractional part of the normalization factor η is constrained as the form of $2^{-n}$ ($n\in N^{*}$) (e.g., 1.5, 1.25, 1.125, etc.), there are not many legal options for this factor. In our work, the value of normalization factor η is taken as 1.125.

In addition, our experiments illustrate that these legal options result in very slight performance differences, as shown in Figure 3 and Figure 4. The optimal factor can be discovered from the experiments that used a 192 code length, as described in this study. However, the greatest advantage of the proposed normalization factor lies in greatly reducing the logic resources in the hardware implementation, while maintaining a similar performance or having a negligible loss of performance. In this view, the factor is independent of code length or code rate to some extent.

**Figure 6:**
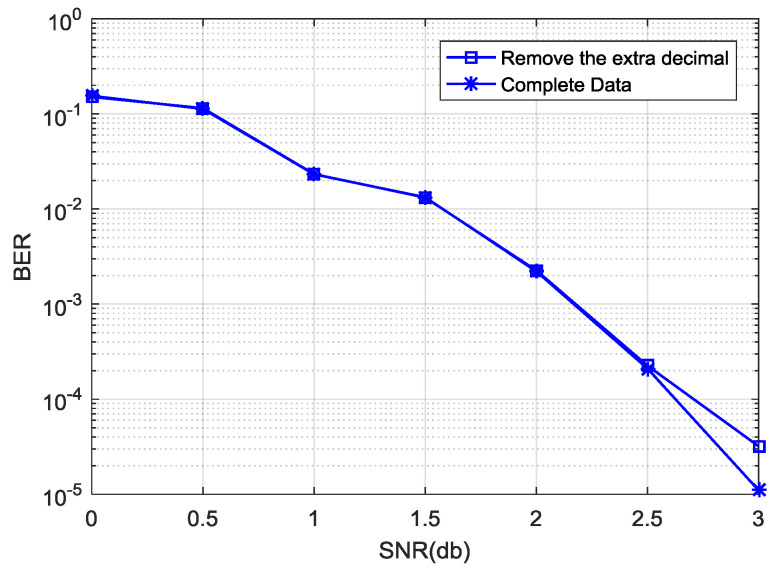
Decoding performance of Nor-Log-MAP in a hardware simulation.

Figure 5 shows the effect of decoding performance under the normalization factor η=1.125 with different |a−b| ranges, where we define |a−b|<range. In Figure 5, the decoding performance is better when range<9 and range<10, and the decoding performance with range<9 is better than that range<10 with a signal noise ratio (SNR) from 1.5 to 2.8 dB. Considering decoding performance with a medium to high SNR, the range |a−b| can be determined as |a−b|<9. Therefore, the Nor-Log-MAP algorithm can be rewritten as Equation (13). In the hardware simulation, Equation (13) can be realized by Equation (14) after binary conversion, where max (a,b)×0.001 can be computed by intercepting the corresponding data based on selected bit width and the number of decimal digits. During the hardware simulation, 7-bit channel information is used, where 5 bits are integers and 2 bits are decimals. When computing, the data part that exceeds two decimal places is removed, however this results in performance loss. As can be seen from Figure 6, there is a little gap between the removed extra decimal places and the complete data in decoding performance. We also know that hard decision in turbo decoding relates to the sign bit of information directly rather than the absolute value of the data. In this way, the algorithm can replace multiplication with an addition operation, and will reduce some logic resource consumption compared with the lookup table method.

(13) $$\max^{*}(a,b)\approx \{\begin{array}{c} 1.125\cdot \max(a,b)\\ \max(a,b) \end{array}\begin{array}{c} ,if|a-b|<9\\ ,\mathrm{otherwise} \end{array}$$

(14) $$\max^{*}(a,b)\approx \{\begin{array}{c} \max(a,b)+\max(a,b)*0.001\\ \max(a,b) \end{array}\begin{array}{c} ,if|a-b|<9\\ ,\mathrm{otherwise} \end{array}$$

**Figure 7:**
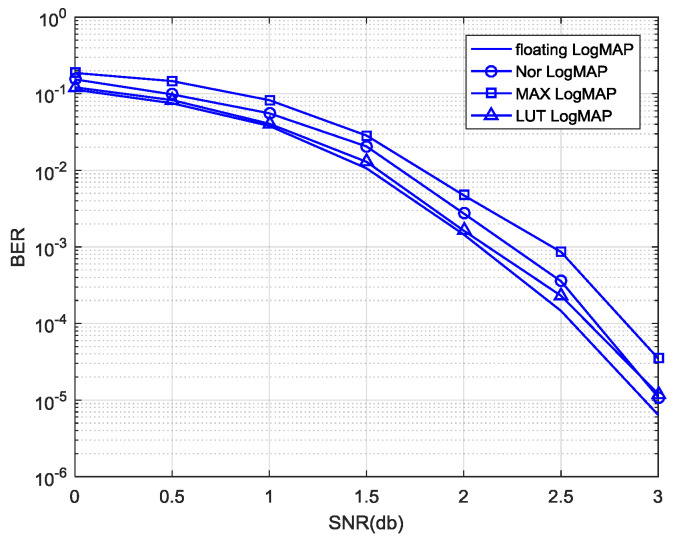
Comparison of different Log-MAP approximate algorithms in decoding performance.

The comparison between the proposed Nor-Log-MAP algorithm and other approximate algorithms in decoding performance is shown in Figure 7. As can be seen, the decoding performance of the proposed Nor-Log-MAP algorithm is superior to the Max-Log-MAP algorithm, and has a maximum gain of 0.25 dB in decoding performance. Compared with the Log-MAP algorithm and LUT-Log-MAP algorithm, the Nor-Log-MAP algorithm has some disadvantages, but the decoding performance of the Nor-Log-MAP algorithm is close to (or even surpasses) the LUT-Log-MAP algorithm with a high SNR. Considering that the logic complexity of the Nor-Log-MAP algorithm is close to that of the Max-Log-MAP algorithm theoretically, it can be concluded that the Nor-Log-MAP algorithm has a considerable decoding performance and less logic complexity.

## 4. The SISO Design of the Turbo Decoder

**Figure 8:**
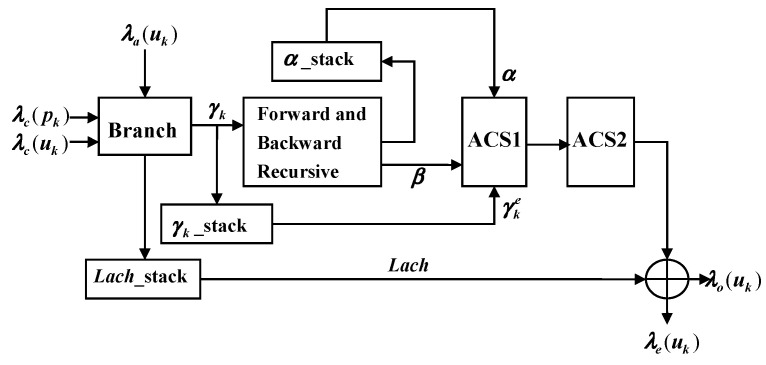
SISO structure of turbo decoder.

The decoding process of turbo codes in SISO is shown in Figure 8 [14,15]. Firstly, received system information $\lambda_{c}\left(u_{k}\right)$, check information $\lambda_{c}\left(p_{k}\right),$ and priori information $\lambda_{a}\left(u_{k}\right)$ are used to compute $\gamma_{k}$, $\gamma_{k}^{e}$, and *Lach* in the branch unit. Then, we use a generated branch metric and initialized value α and to compute the new α and β through a forward and backward recursive (FBR) unit. Finally, add compare select 1 (ACS1) and ACS2 are used to compute posteriori information $\lambda_{o}\left(u_{k}\right)$ and external information $\lambda_{e}\left(u_{k}\right)$ [16,17].

**Figure 9:**
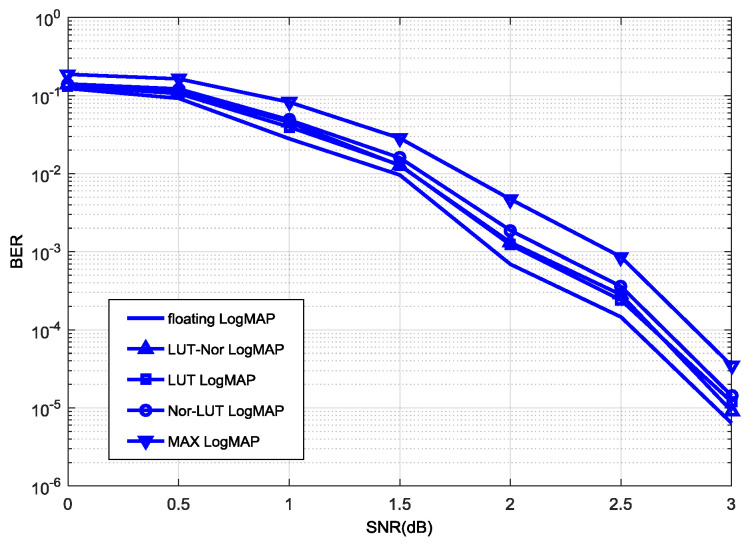
Decoding performance of the LUT-Nor-Log-MAP algorithm.

In Figure 7, the LUT-Log-MAP algorithm is demonstrated to be superior to the Nor-Log-MAP algorithm in decoding performance, so we tried to employ these two algorithms using a turbo decoder for better decoding performance. In the simulation, the LUT-Log-MAP algorithm is adopted in computing forward-state and backward-state metrics, while posteriori information and external information are computed by the Nor-Log-MAP algorithm. The decoding performance of this hybrid algorithm is shown in Figure 9. As can be seen, the LUT-Nor-Log-MAP algorithm not only compensates for the decoding performance of the Nor-Log-MAP algorithm with a low SNR, but also maintains the advantages of the Nor-Log-MAP algorithm with a high SNR. Furthermore, compared to the Max-Log-MAP algorithm, it has maximum of a 0.5 dB increase in decoding performance.

At the same time, we also propose the Nor-LUT-Log-MAP algorithm, which uses the Nor-Log-MAP algorithm to calculate forward-state and backward-state metrics as well as the LUT-Log-MAP algorithm to compute external information and posteriori information. As shown in Figure 9, the decoding performance of the Nor-LUT-Log-MAP algorithm is less than that of the LUT-Nor-Log-MAP algorithm, and it can be concluded that the forward-state and backward-state metric computation requires a better algorithm in the turbo decoding process [18].

**Figure 10:**
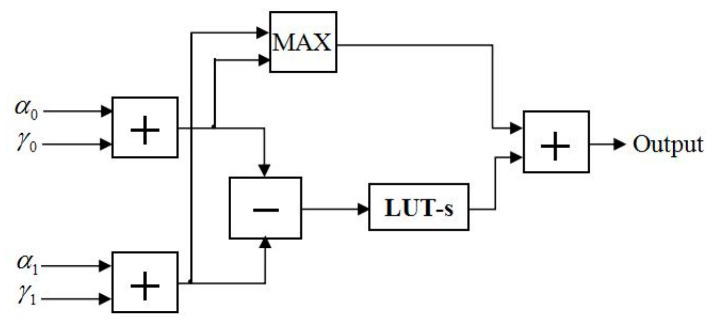
Function module structure of the FFU.

**Figure 11:**
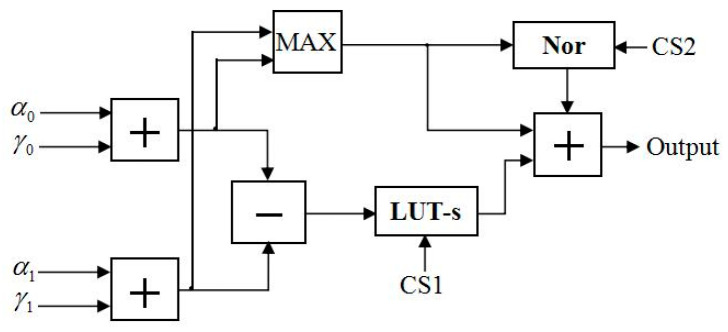
Function module structure of the NFU.

In [10], Sun et al. proposed FFU to compute the metrics of every state. The structure of the FFU is shown in Figure 10, where the input signals of the module are α and γ during the forward-state metric computation. In order to satisfy the proposed LUT-Nor-Log-MAP algorithm, we designed an NFU based on the FFU, which is applicable to all state metric calculation at the same time. The structure of the NFU is shown in Figure 11. The operation of the lookup table unit and the normalization unit are controlled by control signals (CSs). LUT-s and *Nor* represent lookup table approximation and the normalized approximation for function $\log(1+e^{-\left|a-b\right|})$, respectively. Hence, it is possible to adopt different approximation methods for satisfying the forward-state and backward-state metrics, as well as posteriori information and external information computation.

**Figure 12:**
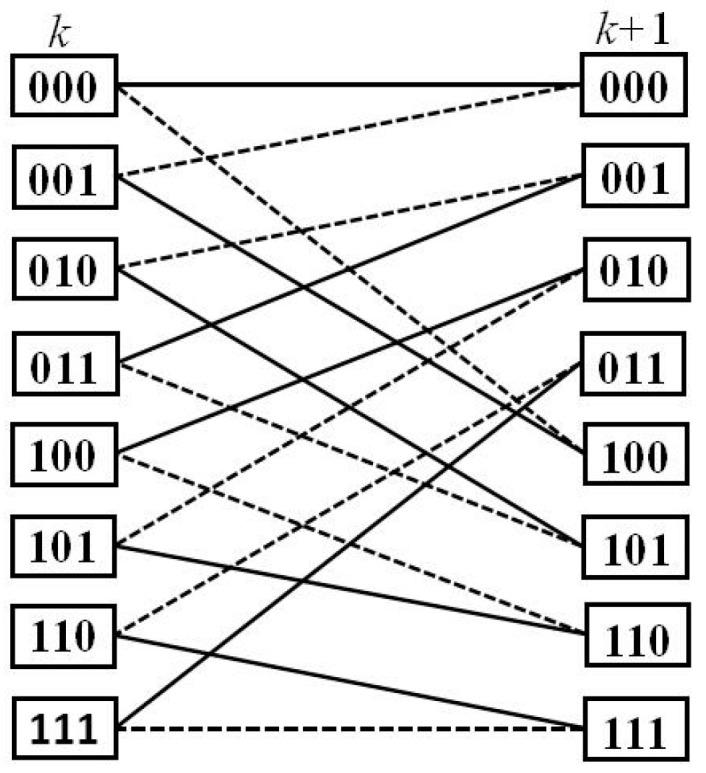
The rule of state trellis transition for the TD-LTE turbo codes.

**Figure 13:**
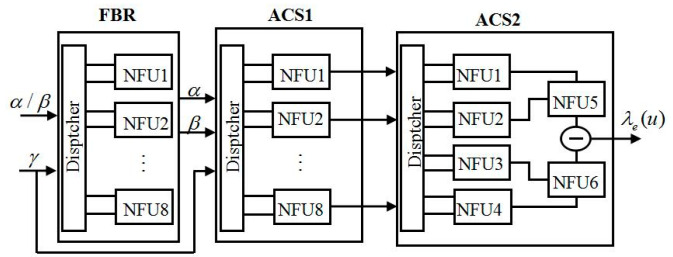
Structure of the modified SISO unit.

Figure 12 shows the rule of the state trellis transition of the turbo decoder under the time division long term evolution (TD-LTE) standard. In order to satisfy the eight-state transition rule of the turbo trellis network in the decoding process [19,20], the FBR unit calls eight NFUs, and the ACS1 and ACS2 units call eight and six NFUs, respectively. The structure of the modified SISO unit is shown in Figure 13. Each NFU will complete the calculation of max* one at a time. As can be seen from Figure 11 and Figure 13, for the eight-state turbo code with TD-LTE standard, a codeword for each turbo code needs at least 16 LUTs for the calculation of forward-state and backward-state metrics [21], and an external or posteriori information bit requires 14 LUTs for max* operation. As the degree of parallelism increases, the number of LUTs increase with the same multiple. Therefore, in hardware implementation, the proposed LUT-Nor-Log-MAP algorithm can transform the required 14 LUTs operation per bit into addition operations during turbo code decoding, and thus reduce logic resource consumption.

Table 3 shows the logic resource consumption of the turbo decoder and its SISO under different algorithms. Simulation results show that decoder logic resource consumption based on the LUT-Nor-Log-MAP algorithm is 74.5 K. Compared with the Max-Log-MAP algorithm, our design consumes more about 8.4% logic resources, but our design has advantages in decoding performance. Compared with the LUT-Log-MAP algorithm, our design saves about 2.1% of logic resources with a similar decoding performance.

## 5. Complexity Analysis of the Proposed Algorithm

In the improved Log-MAP algorithm, the main part of improvement is the $\log(1+e^{-\left|a-b\right|})$ of the function max*, so that different algorithms have different complexities of the max* function. The complexity analysis of this section starts with one decoded bit (or a trellis transition), then we calculate the complexity of the Log-MAP algorithm and the complexity of the function max* using different approximation algorithms [22,23].

First of all, for the rule of an eight-state trellis transition of the TD-LTE turbo codes, there are 16 branch metrics, which means that there are 16 message transmission paths. The complexity calculation of the Log-MAP algorithm is as follows:

Step 1: The calculation of the branch metric by Equation (2) requires a total of 8 × 2 × n addition operations and 8 × 2 × n multiplication operations.

Step 2: In Equation (4), the calculation of forward-state metrics requires a total of eight additions, which correspond to the forward-state metrics of the eight sides in Figure 12, and this requires eight max* calculations.

Step 3: Calculation of backward-state metrics using Equation (5) is the same as in Step 2.

Step 4: In Equation (6), the calculation of external information needs 8 × 4 additions and one subtraction operation; it also requires a total of 8 + 4 + 2 max* calculations in hardware implementation. The logic addition is same as subtraction.

Step 5: The calculation of posteriori information only requires 8 × 2 additions in Equation (7).

The complexity analysis of the Log-MAP algorithm is summarized in Table 4. As can be seen, the total complexity of the Log-MAP algorithm is 95 + 32 × n, which consists of 65 + 16 × n additions or subtractions, 16 × n multiplications, and 30 max* operations.

Next, the different complexities of approximation algorithms of the function max* are analyzed as follows. As can be seen in Figure 10, only two additions and one comparison are performed for Max-Log-MAP algorithm, due to the omission of the $\log(1+e^{-\left|a-b\right|})$ of the function max*. For the LUT-Log-MAP algorithm, two additions are made at first, followed by a comparison and a subtraction, then the LUT equivalent to four comparators. Finally, the results of the lookup table make an addition with the comparison results of the function max. As can be seen in Figure 11, for the Nor-Log-MAP algorithm, two additions are performed at first, then the results go through a normalization operation after the function max comparison; for the selected normalized factor in Equation (14), every datum needs to be shifted three times. Finally, an addition operation is used to sum. In addition to considering the complexity of the LUT-Log-MAP and Nor-Log-MAP algorithms, the LUT-Nor-Log-MAP algorithm requires a mode comparison to choose between LUT-Log-MAP decoding and Nor-Log-MAP decoding. The synthesis results of the complexity for different Log-MAP approximation algorithms are shown in Table 5.

We can see from Table 5 that the Max-Log-MAP algorithm only needs two additions and one comparison in the max* function, but its decoding performance is the worst. Compared with the LUT-Log-MAP algorithm, the Nor-Log-MAP algorithm reduces a subtraction and four comparisons in the max* function, but increases three shifts. According to Table 3, it can be seen that Nor approximation reduces consumption of logic resources compared with LUT approximation. By adding a mode comparison, the LUT-Nor-Log-MAP algorithm can be switched between LUT and Nor approximation in function max* processing. The simulation results of Table 3 show that the LUT-Nor-Log-MAP algorithm consumes more logic resources than the Nor-Log-MAP algorithm, which is in line with expectations. However, compared to the LUT-Log-MAP algorithm, the proposed algorithm still reduces logic resource consumption considerably.

## 6. Synthesis Results and Comparisons

In this paper, a turbo decoder was designed using the Cyclone IV series FPGA EP4CE115F29C7 chip as the target device, and the QPP interleaver was used for interleaving and de-interleaving. In simulation, we adopted turbo codes with a 3072 information block size and 1/2 code rate, and allocated 12 SISO decoders to work in parallel with a maximum clock frequency *M* of 44 MHz. Through Equation (15), we can see that the throughput of the proposed decoder is 36 Mbit/s when the clock cycle of each iteration is 1502 and the maximum number *N* of iterations is 5.

(15) $$\mathrm{Throughput}=\frac{\mathrm{Code}\;\mathrm{Length}\times M}{\mathrm{Clock}\;\mathrm{Cycle}\times N}$$

The comparisons with other literatures are presented in Table 6. As can be seen, the LUT-Nor-Log-MAP algorithm achieved a decoding throughput of 36 Mbit/s in five iterations. Compared with [24], we had advantages in decoding performance and logic resource consumption, and under the same clock frequency our design was able to achieve a higher throughput. Compared with [25], our algorithm had obvious advantages in throughput. Although the throughput of our design was close to [26] and less than [27], the proposed LUT-Nor-Log-MAP algorithm had better decoding performance than the Max-Log-MAP algorithm [26]. Compared with [27], the logic resource consumption in our design was greatly reduced.

## 7. Conclusions

Future wireless communication standards will require a turbo decoder with higher throughput and better decoding performance. In this paper, we proposed a turbo decoder based on the LUT-Nor-Log-MAP algorithm applied to all kind of turbo codes under the TD-LTE wireless communication standard. For turbo codes under other standards, we could follow the same improvement idea to optimize the performance of the decoder. The proposed decoder was based on the Cyclone platform, in which clock frequency is low, so we could not get a higher throughput directly. Based on the proposed Nor-Log-MAP algorithm, we tried to achieve a better decoding performance with the medium and low SNR. Compared with the Max-Log-MAP and LUT-Log-MAP algorithms, the proposed LUT-Nor-Log-MAP algorithm not only guaranteed considerable decoding performance but also reduced logic resource consumption. Considering that the SISO decoder based on the NFU in actual design can save about 15% of logic resources compared with the FFU, the LUT-Nor-Log-MAP algorithm could reduce more consumption of logic resources than the LUT-Log-MAP algorithm in turbo decoder design.

## Figures and Tables

**Table 1:** The max* approximation method for a lookup table.

|a−b|	|a−b|=0	0<|a−b|≤0.75	0.75<|a−b|≤2	|a−b|>2
$\log(1+e^{-|a-b|})$	0.69	0.5	0.25	0

**Table 2:** The lookup-table method based on Q2 quantization.

|a−b|	|a−b|=0	0<|a−b|≤3	3<|a−b|≤8	|a−b|>8
$\log(1+e^{-|a-b|})$	3	2	1	0

**Table 3:** Simulation results of logic resource consumption.

Algorithm	LUT-Log-MAP	Max-Log-MAP	Nor-Log-MAP	LUT-Nor-Log-MAP
Decoder Gate Count(K)	76.1 K	68.7 K	71.8 K	74.5 K
SISO Gate Count(K)	4.44 K	3.65 K	4.02 K	4.32 K

**Table 4:** Complexity analysis of the Log-MAP algorithm.

Algorithm	ADD/SUB	Multiplication	Max *
Step1 ($\gamma_{k}$)	8 × 2 × n	8*2*n	0
Step2 ($\alpha_{k}\left(s_{k}\right)$)	8	0	8
Step3 ($\beta_{k}\left(s_{k}\right)$)	8	0	8
Step4 ($\lambda_{e}\left(u_{k}\right)$)	8 × 2 × 2 + 1	0	8 + 4 + 2
Step5 ($\lambda_{o}\left(u_{k}\right)$)	8*2	0	0
all	65 + 16 *n	16 *n	30

**Table 5:** Simulation results and comparison.

Algorithm	ADD	SUB	Comparison	Shift
Max	2	0	1	0
LUT	2	1	(4 + 1)	0
Nor	2	0	1	3
LUT-Nor	2	1 or 0	(4 + 1 + 1) or 2	0 or 3

**Table 6:** Simulation results and comparison.

	This Work	[24]	[26]	[27]	[25]
Code Standard	3GPP-LTE	3GPP-LTE	DVB-SH	3GPP-LTE	3GPP-LTE
Implementation	Cyclone IV	TTA	ASIC	CMOS	ASIP
Algorithm (Log-MAP)	LUT-Nor	Max	Max	LUT	Max
Code Length	6144	6144	6144	4096	6144
Iterations	5	6	NA	6	5
Gate Count (K)	74.5	85.7	50	900	NA
Clock Frequency (MHz)	44	210	346	252	200
Throughput (Mbit/s)	36	65.1	346	535	22
